# Supplementary figures and images for: The genetic architecture of resistance to flubendiamide insecticide in Helicoverpa armigera (Hübner)
Source: PLoS One. 2025 Jan 29;20(1):e0318154. doi: 10.1371/journal.pone.0318154 (PMC11778771; doi:10.1371/journal.pone.0318154)

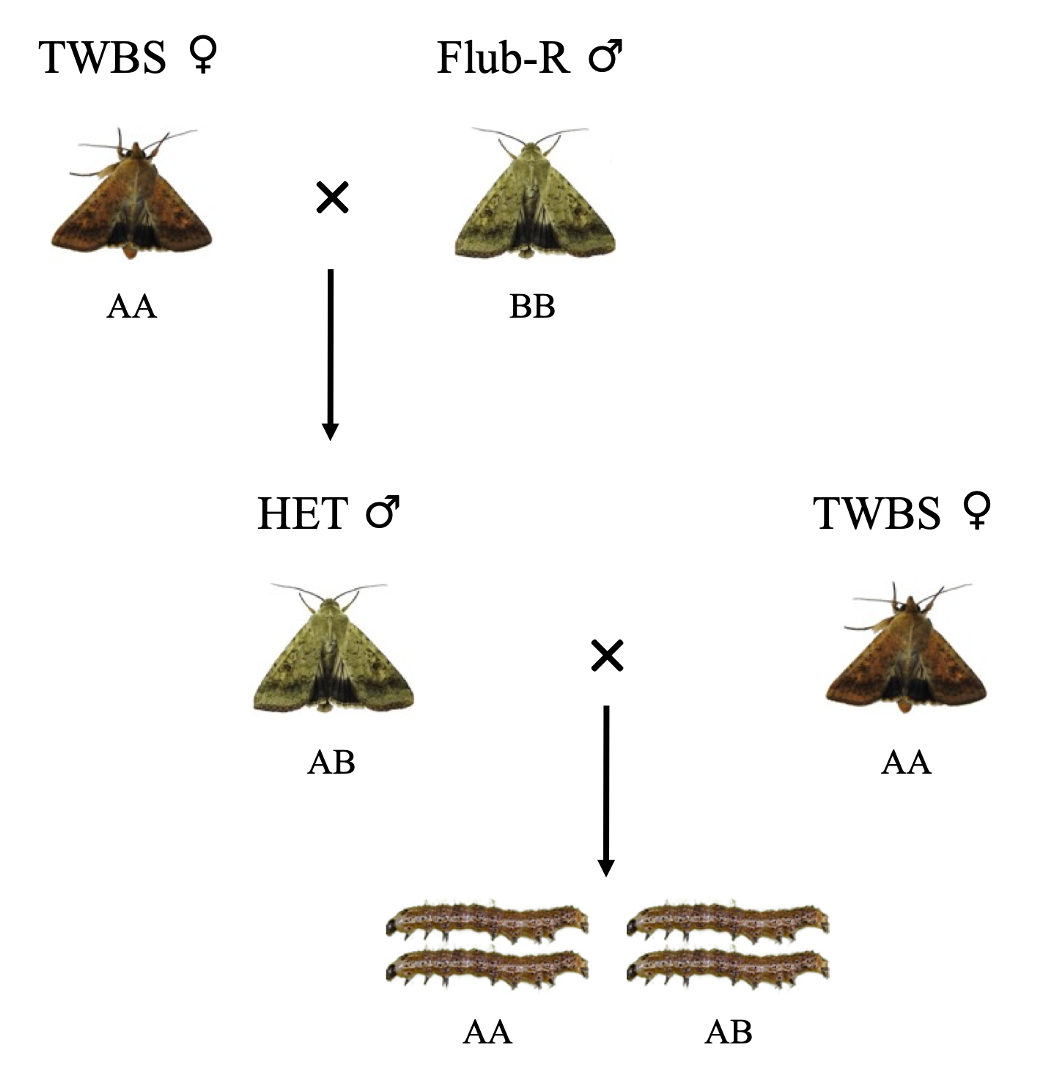

Supplement: S1 Fig — The backcross population originated from the cross between the Helicoverpa armigera strains Flub-R (Resistant) and TWBS (Susceptible). The larvae represent the individuals used for DNA sequencing using the GBS method. The AA code denotes the homozygous susceptible, the BB homozygous resistant and AB the heterozygous. (TIF) [file pone.0318154.s001.tif]

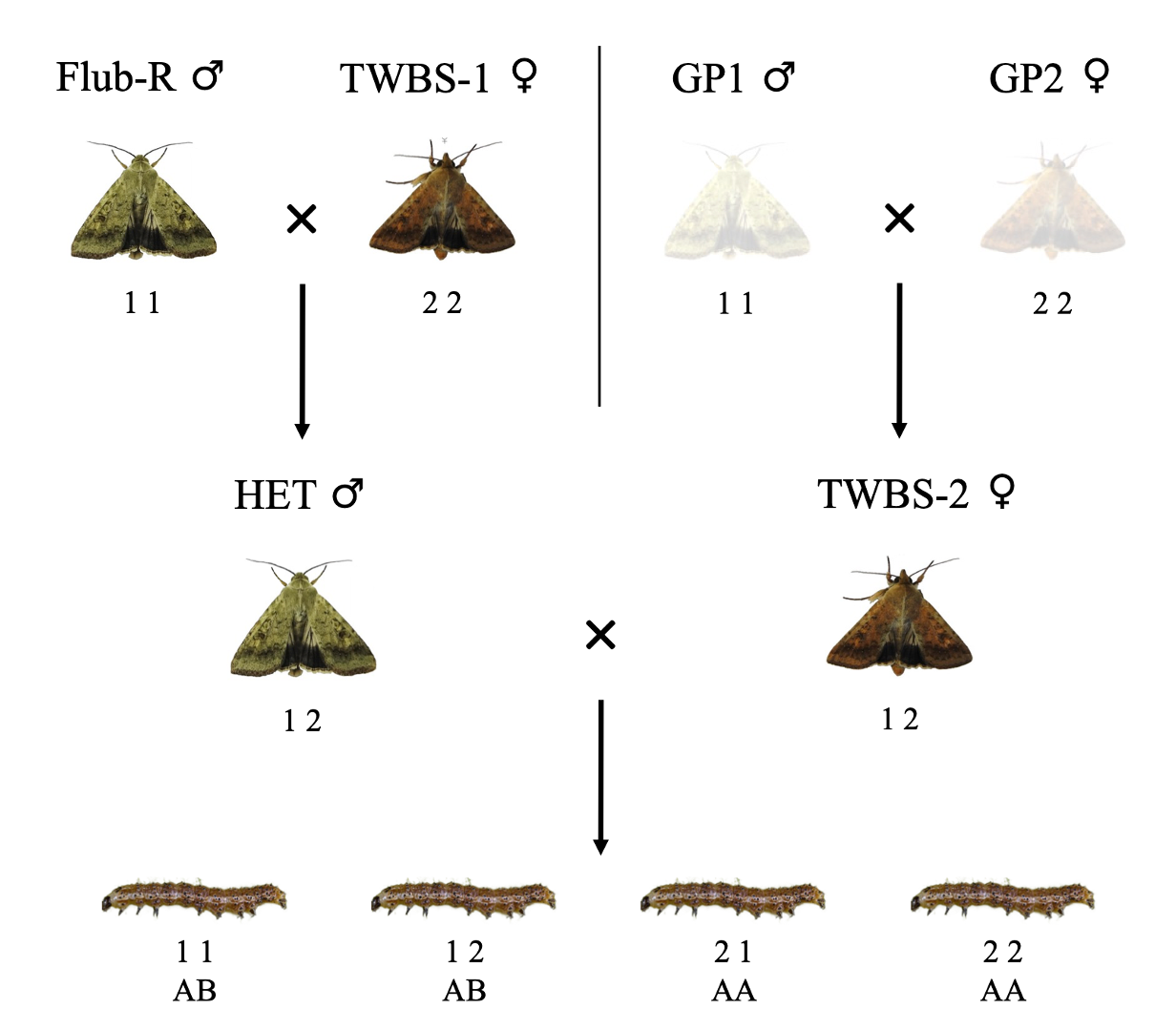

Supplement: S2 Fig — Grandparents 1 and 2, shown as semi-transparent, represent dummy grandparents that were added to the pedigree file of the BC1 population, as indicated by the LepMap3 manual. The values represent the genotype codes used by the programme, where 11 and 22 denote male homozygotes and female homozygotes, respectively. The codes 12 and 21 represent heterozygotes. The first number originates from the paternal side, and the second is from the maternal side. These numeric codes were converted to AA and AB codes used by the rQTL programme, representing susceptible homozygotes and heterozygotes, respectively. (TIF) [file pone.0318154.s002.tif]

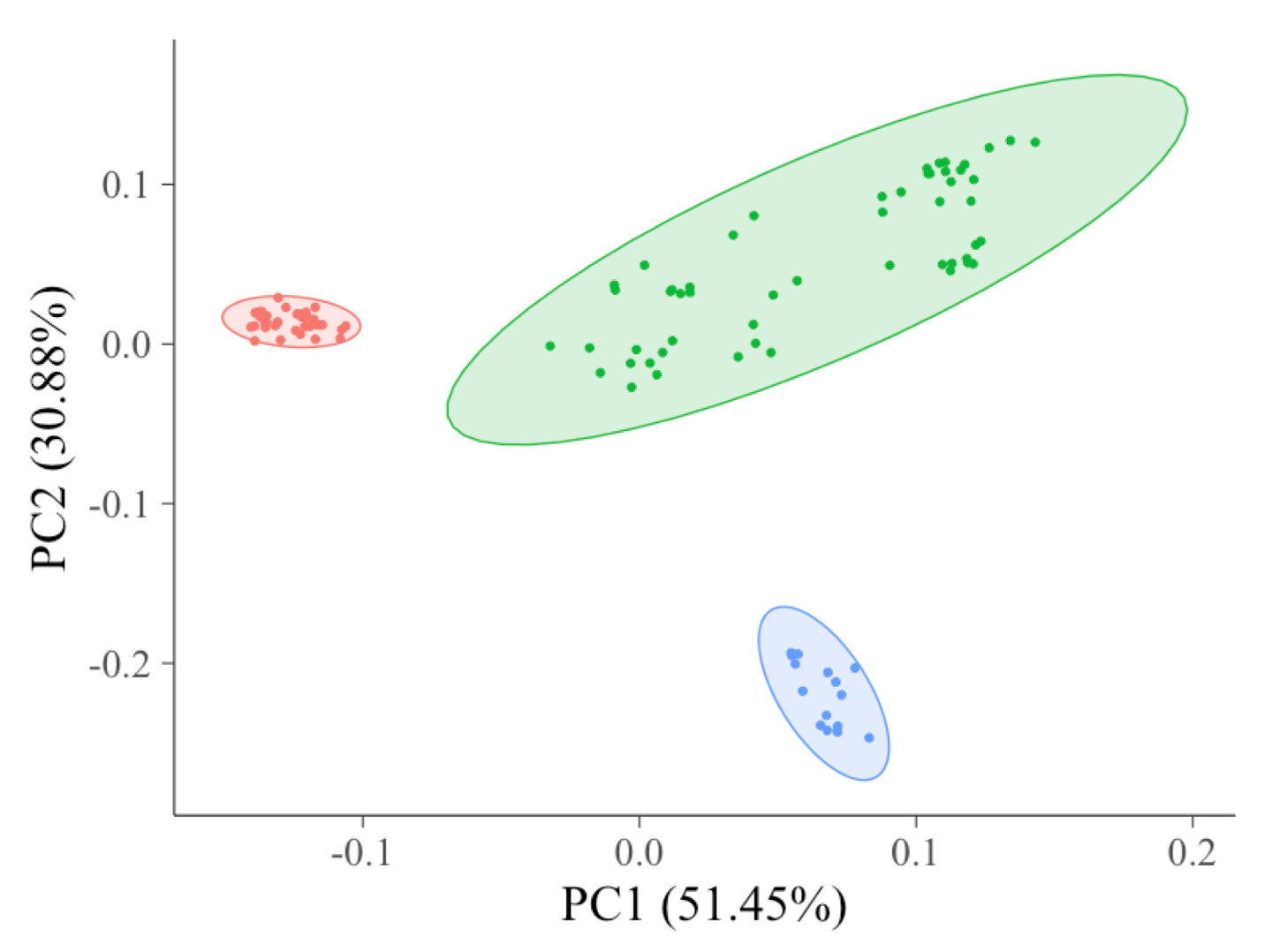

Supplement: S3 Fig — The colours represent the sample clusters identified by PCA analysis, indicating K = 3. The circles denote the Euclidean distance from the centre of each cluster, corresponding to the 95% confidence ellipse. (TIF) [file pone.0318154.s003.tif]

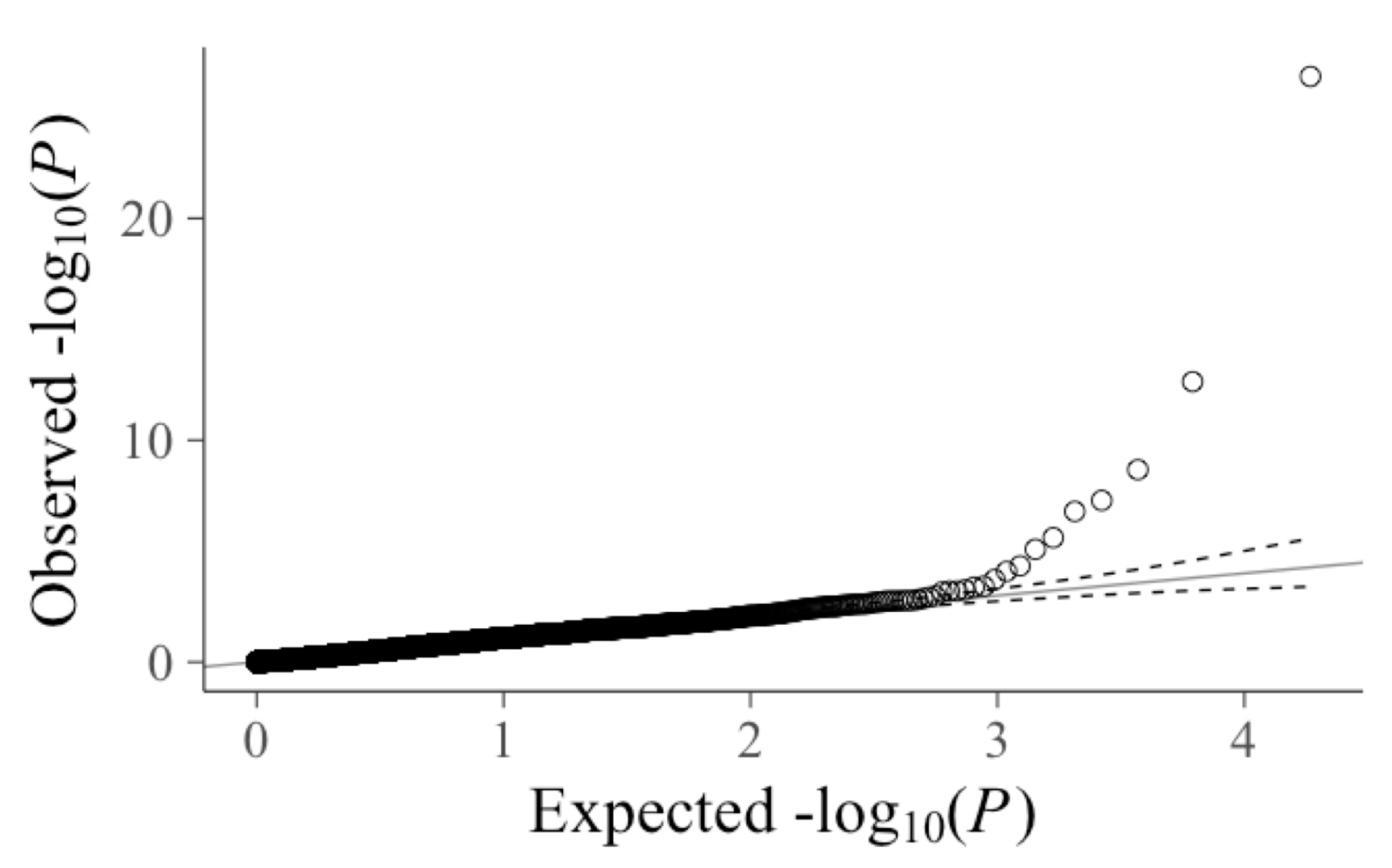

Supplement: S4 Fig — The light grey line shows the −log10(p- values) expected. The dashed lines represent the upper and lower limits of the 95% confidence interval. The black unfilled circles show the −log10(p- values) observed. (TIF) [file pone.0318154.s004.tif]

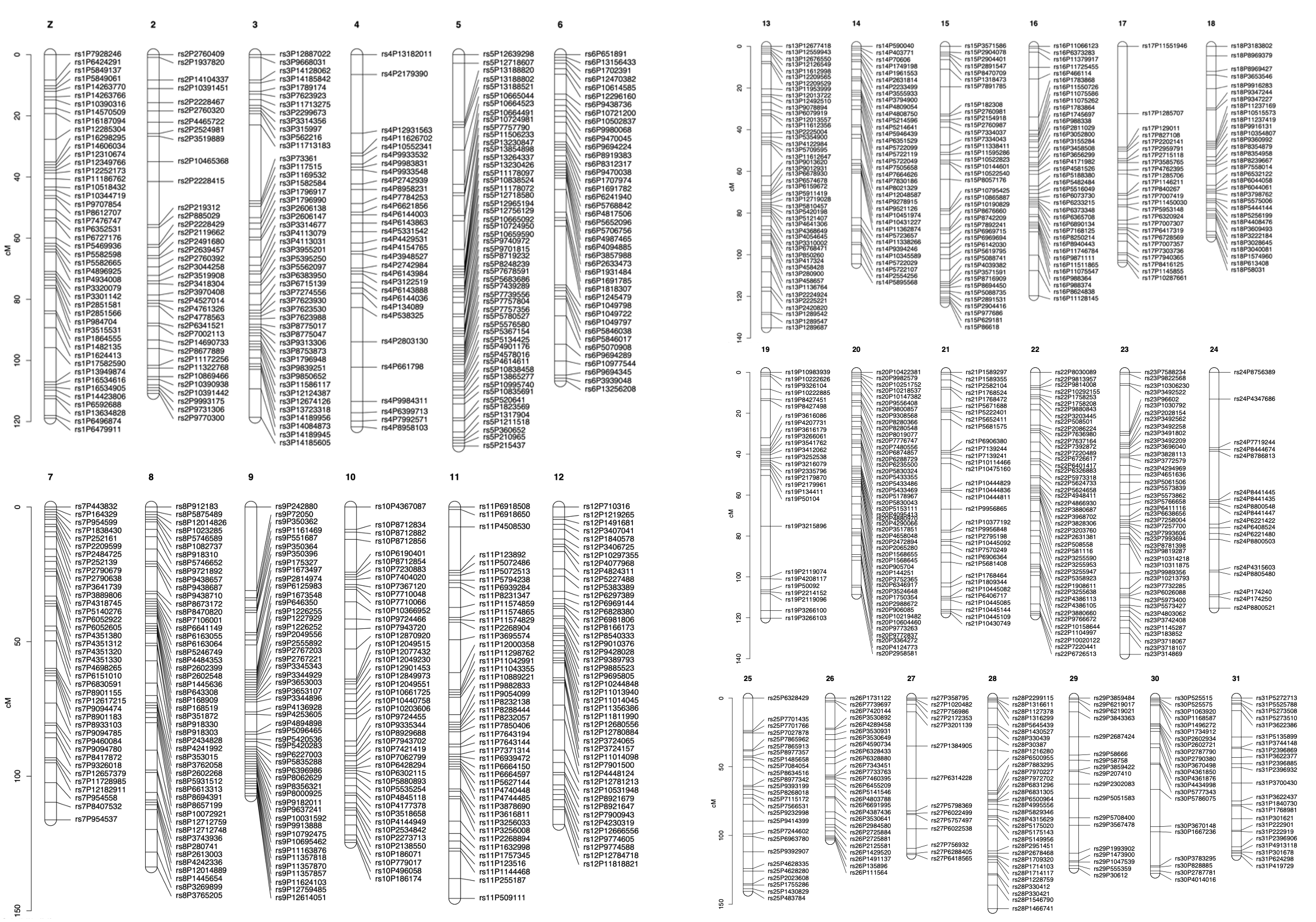

Supplement: S5 Fig — Each line in a linkage group denotes the position of a marker, with its respective name beside it. The y-axis shows the markers’ genetic positions and the linkage groups’ total size in centiMorgans (cM). (TIF) [file pone.0318154.s005.tif]
