# Supplementary material for: The genetic architecture of resistance to flubendiamide insecticide in Helicoverpa armigera (Hübner)
Source: PLoS One. 2025 Jan 29;20(1):e0318154. doi: 10.1371/journal.pone.0318154 (PMC11778771; doi:10.1371/journal.pone.0318154)
Supplement: S1 Table — (PDF) [file pone.0318154.s006.pdf]

S1 Table. Linkage map summary.

LG	Nº. Markers	Length (cM)	Mean Spacing (cM)	Max Spacing (cM)
Z	46	118.94	2.64	11.19
2	37	110.76	3.08	14.13
3	47	118.63	2.58	24.78
4	31	121.68	4.06	19.25
5	52	127.96	2.51	19.25
6	41	106.68	2.67	19.25
7	39	116.21	3.06	10.23
8	49	133.70	2.79	10.23
9	50	107.31	2.19	30.95
10	45	124.58	2.83	12.16
11	45	145.38	3.30	16.13
12	45	117.88	2.68	11.19
13	46	135.06	3.00	15.12
14	36	104.26	2.98	21.40
15	40	123.02	3.15	14.13
16	38	119.74	3.24	18.19
17	25	103.66	4.32	32.29
18	32	91.56	2.95	15.12
19	27	120.22	4.62	24.79
20	47	108.65	2.36	12.16
21	33	117.73	3.68	15.12
22	44	118.71	2.76	8.33
23	43	137.88	3.28	22.51
24	18	115.27	6.78	23.64
25	26	140.68	5.63	23.63
26	27	103.16	3.97	19.25
27	14	113.88	8.76	24.78
28	32	153.26	4.94	18.19
29	18	123.98	7.29	20.32
30	22	127.62	6.08	27.16
31	23	114.71	5.21	19.25
