# Supplementary material for: The genetic architecture of resistance to flubendiamide insecticide in Helicoverpa armigera (Hübner)
Source: PLoS One. 2025 Jan 29;20(1):e0318154. doi: 10.1371/journal.pone.0318154 (PMC11778771; doi:10.1371/journal.pone.0318154)
Supplement: S2 Table — (PDF) [file pone.0318154.s007.pdf]

S2 Table. List of genes within a 300 Kb downstream and upstream from the markers identified by GWAS and QTL mapping, linked to *H. armigera* survival to flubendiamide.

Chromosome	RefSeq	Start	End	Strand	Gene ID	Description	Associated SNPs (Statistical Analysis)
Z	NC_087120.1	3156825	3168521	+	LOC110379460	uncharacterized protein CG1161	
Z	NC_087120.1	3169578	3182738	-	LOC110379465	uncharacterized LOC110379465	
Z	NC_087120.1	3183368	3217595	+	LOC110379468	roquin-1	
Z	NC_087120.1	3224958	3247304	+	LOC110379469	uncharacterized LOC110379469	
Z	NC_087120.1	3250802	3259898	+	LOC110373007	atypical kinase COQ8B-2C mitochondrial	
Z	NC_087120.1	3268500	3278214	+	Wrm1	wurmchen 1	
Z	NC_087120.1	3271193	3277075	-	LOC135118117	uncharacterized LOC135118117	
Z	NC_087120.1	3280642	3281665	-	LOC110373795	peptidyl-prolyl cis-trans isomerase-like 1	
Z	NC_087120.1	3282086	3303330	+	LOC110373753	rho GTPase-activating protein 1	
Z	NC_087120.1	3305424	3349850	+	LOC110373799	PHD finger protein rhinoceros	
Z	NC_087120.1	3360832	3475315	+	Nwk	nervou swreck	rs1P3460302 (GWAS)
Z	NC_087120.1	3481796	3492756	-	LOC110379461	atypical kinase COQ8B-2C mitochondrial	
Z	NC_087120.1	3501037	3517277	-	LOC110373023	Phosphatidylserine lipase ABHD16A	
Z	NC_087120.1	3518186	3536343	+	LOC110373032	protein GPR107	
Z	NC_087120.1	3552592	3579679	+	LOC110373030	ras-relatedandestrogen-regulated growth inhibitor	
Z	NC_087120.1	3583124	3593025	+	LOC110373024	nuclear receptor subfamily 2 group E member 1	
Z	NC_087120.1	3597157	3649026	+	LOC110373000	calcium-binding mitochondrial carrier protein SCaMC-2	
Z	NC_087120.1	3653786	3665298	-	LOC110373012	lysine-specific demethylase 3A-A	
Z	NC_087120.1	3667365	3683354	+	LOC110372998	ATP-binding cassette sub-family F member 2	
Z	NC_087120.1	3683355	3902488	-	LOC126054100	uncharacterized LOC126054100	
Z	NC_087120.1	3750422	3750495	+	Trnai-aau	transfer RNA isoleucine (anticodon AAU)	
2	NC_087121.1	1634694	1637974	+	LOC110376521	lipase member H	
2	NC_087121.1	1660167	1661511	-	LOC110376516	lipase member H	
2	NC_087121.1	1663158	1671650	-	LOC110376523	lipase member I	
2	NC_087121.1	1666802	1670124	-	LOC110376524	lipase member H	
2	NC_087121.1	1690778	1694332	-	LOC135118694	uncharacterized LOC135118694	
2	NC_087121.1	1701866	1703839	-	LOC126055277	uncharacterized LOC126055277	
2	NC_087121.1	1713506	1713577	-	Trnaa-cgc-3	transfer RNA alanine (anticodon CGC)	
2	NC_087121.1	1786763	1788331	+	LOC110376529	uncharacterized LOC110376529	
2	NC_087121.1	1806428	1820115	+	LOC110376514	uncharacterized LOC110376514	
2	NC_087121.1	1840635	1941818	+	LOC110372095	uncharacterized LOC110372095	rs2P1937820 (Lower boundary of the QTL)
2	NC_087121.1	1943506	1945479	+	LOC126056517	uncharacterized LOC126056517	
2	NC_087121.1	2023799	2023870	-	Trnad-guc-3	transfer RNA aspartic acid (anticodon GUC)	
2	NC_087121.1	2026439	2027764	+	LOC126055600	uncharacterized LOC126055600	
2	NC_087121.1	2027222	2028898	-	LOC126055594	uncharacterized LOC126055594	
2	NC_087121.1	2048721	2059694	-	LOC110372073	probable 3-hydroxyisobutyrate dehydrogenase%2C mitochondrial	
2	NC_087121.1	2065610	2074390	-	LOC110372049	sulfotransferase 1E1	
2	NC_087121.1	2092901	2106319	+	LOC110372097	sulfotransferase 1C3	
2	NC_087121.1	2114207	2193215	-	LOC110372027	protein tweety	
2	NC_087121.1	2140556	2147706	+	LOC126054659	uncharacterized LOC126054659	
2	NC_087121.1	2178498	2180448	+	LOC126054004	uncharacterized LOC126054004	
2	NC_087121.1	2180586	2182469	-	LOC135117689	putative nuclease HARBI1	
2	NC_087121.1	2193487	2195705	+	LOC110372086	cytosolic Fe-S cluster assembly factor Nubp2 homolog	
2	NC_087121.1	2196121	2215720	-	LOC110372048	cryptochrome-1	
2	NC_087121.1	2215819	2216848	+	LOC110372042	uncharacterized LOC110372042	
2	NC_087121.1	2218022	2227209	+	LOC110372089	uncharacterized LOC110372089	
2	NC_087121.1	2227169	2236910	-	LOC110372088	uncharacterized LOC110372088	

2	NC_087121.1	2458435	2463133	-	LOC110372053	developmentally-regulated GTP-binding protein 2	
2	NC_087121.1	2464426	2465938	+	LOC110372072	uncharacterized LOC110372072	
2	NC_087121.1	2466302	2471710	+	LOC110372077	probable RNA-binding protein 46	
2	NC_087121.1	2471880	2480711	+	LOC110372076	uncharacterized LOC110372076	
2	NC_087121.1	2489368	2565140	-	Myo10a	Myosin 10A	
2	NC_087121.1	2566739	2567563	+	LOC110372060	protein BUD31 homolog	
2	NC_087121.1	2568025	2571380	-	LOC110372059	innexin inx2	
2	NC_087121.1	2572045	2596108	-	LOC110372057	alkyldihydroxyacetonephosphate synthase	
2	NC_087121.1	2596839	2647954	-	LOC110372051	uncharacterized LOC110372051	
2	NC_087121.1	2664567	2665685	+	LOC110372085	protein transport protein SEC31	
2	NC_087121.1	2667212	2671132	+	LOC110372078	E3 ubiquitin-protein ligase RING1	
2	NC_087121.1	2675090	2676281	+	LOC110372079	large ribosomal subunit protein eL22	
2	NC_087121.1	2677399	2697202	-	LOC110372026	probable aconitate hydratase, mitochondrial	
2	NC_087121.1	2697648	2705986	+	Sec15	Secretory 15	
2	NC_087121.1	2723805	2727015	-	LOC110372061	fatty acid-binding protein, liver	
2	NC_087121.1	2732066	2761175	+	LOC110372044	uncharacterized LOC110372044	rs2P2759433 (GWAS) and rs2P2760409 (QTL Peak)
2	NC_087121.1	2761147	2804657	-	LOC110372045	adenylosuccinate lyase	
2	NC_087121.1	2765810	2801220	+	LOC110372047	uncharacterized LOC110372047	
2	NC_087121.1	2805181	2814541	-	LOC110372043	hydroxysteroid dehydrogenase-like protein 2	
2	NC_087121.1	2818825	2884188	-	Wake	wide awake	
2	NC_087121.1	2890476	2891159	+	LOC135117725	uncharacterized LOC135117725	
2	NC_087121.1	2923606	3082866	-	LOC135118708	uncharacterized LOC135118708	
2	NC_087121.1	2998356	3003561	-	LOC135117926	uncharacterized LOC135117926	
2	NC_087121.1	3005268	3006966	+	LOC110372036	uncharacterized LOC110372036	
2	NC_087121.1	3473236	3479522	+	LOC110372068	uncharacterized LOC110372068	
2	NC_087121.1	3481409	3484180	-	LOC110372069	heparan sulfate glucosamine 3-O-sulfotransferase 3A1	
2	NC_087121.1	3484581	3485938	+	LOC110372071	uncharacterized LOC110372071	
2	NC_087121.1	3486097	3488533	-	LOC110372070	hydroxyacylglutathione hydrolase, mitochondrial	
2	NC_087121.1	3488650	3500367	+	LOC110372067	small subunit processome component 20 homolog	
2	NC_087121.1	3502410	3548390	-	LOC110372075	uncharacterized LOC110372075	
2	NC_087121.1	3549545	3558695	+	LOC110372052	histone-binding protein N1/N2	
2	NC_087121.1	3558978	3560164	-	LOC110372083	prostaglandin reductase 1	
2	NC_087121.1	3564669	3565806	-	LOC110372056	prostaglandin reductase 1	
2	NC_087121.1	3567498	3568599	-	LOC110372096	prostaglandin reductase 1	
2	NC_087121.1	3571882	3572989	-	LOC110375835	prostaglandin reductase 1	
2	NC_087121.1	3576958	3578062	-	LOC126053453	prostaglandin reductase 1	
2	NC_087121.1	3585598	3586701	-	LOC126055293	prostaglandin reductase 1	
2	NC_087121.1	3589539	3610946	+	LOC110375870	uncharacterized LOC110375870	
2	NC_087121.1	3590632	3591773	-	LOC135116681	prostaglandin reductase 1-like	
2	NC_087121.1	3592546	3594011	-	LOC110375848	prostaglandin reductase 1	
2	NC_087121.1	3594503	3595653	-	LOC110375828	prostaglandin reductase 1	
2	NC_087121.1	3598844	3599990	-	LOC135118795	prostaglandin reductase 1-like	
2	NC_087121.1	3603509	3604670	-	LOC135117746	prostaglandin reductase 1-like	
2	NC_087121.1	3606513	3607630	-	LOC110375859	prostaglandin reductase 1	
2	NC_087121.1	3611602	3619942	-	LOC110377250	neprilysin-4	
2	NC_087121.1	3620027	3624803	+	LOC110377256	uncharacterized LOC110377256	
2	NC_087121.1	3625644	3633453	+	LOC110376600	E3 ubiquitin-protein ligase CHIP	
2	NC_087121.1	3634549	3635392	-	LOC135117752	uncharacterized LOC135117752	
2	NC_087121.1	3642556	3755153	+	LOC110375503	uncharacterized protein	

2	NC_087121.1	3726561	3728044	+	LOC135118859	uncharacterized LOC135118859	
2	NC_087121.1	3728111	3731254	+	LOC126054309	uncharacterized LOC126054309	
2	NC_087121.1	3756360	3775657	-	LOC110376418	protein CLEC16A homolog	
2	NC_087121.1	3777311	3864707	-	LOC110377735	uncharacterized LOC110377735	rs2P3779183 (GWAS)
2	NC_087121.1	3877409	3879356	-	LOC110377162	uncharacterized LOC110377162	
2	NC_087121.1	3880914	3884237	+	LOC110376954	apyrase	
2	NC_087121.1	3884593	3896675	-	LOC110376365	mitochondrial glycine transporter A	
2	NC_087121.1	3900453	3911255	-	LOC110375876	myophilin	
2	NC_087121.1	3914760	3915758	-	LOC110376754	uncharacterized LOC110376754	
2	NC_087121.1	3916487	3918276	-	LOC110377188	uncharacterized LOC110377188	
2	NC_087121.1	3918961	3924593	-	LOC110376877	uncharacterized LOC110376877	
2	NC_087121.1	3926571	3928731	+	LOC110376964	alpha-(1%2C3)-fucosyltransferase C	
2	NC_087121.1	3928922	3930605	-	LOC110376868	alpha-(1%2C3)-fucosyltransferase C	
2	NC_087121.1	3932414	3933628	-	LOC126056580	uncharacterized LOC126056580	
2	NC_087121.1	3936031	3938034	+	LOC126055286	alpha-(1%2C3)-fucosyltransferase C	
2	NC_087121.1	3940695	3942903	+	LOC135116619	alpha-(1%2C3)-fucosyltransferase C-like	
2	NC_087121.1	3944195	3946368	+	LOC110377087	alpha-(1%2C3)-fucosyltransferase C	
2	NC_087121.1	3946574	3949869	+	LOC110377096	alpha-(1%2C3)-fucosyltransferase C	
2	NC_087121.1	3963051	3965022	+	LOC110377152	uncharacterized LOC110377152	
2	NC_087121.1	3965236	3966796	+	LOC110375483	uncharacterized LOC110375483	
2	NC_087121.1	3967009	3968680	-	LOC110375478	uncharacterized LOC110375478	
2	NC_087121.1	3970104	3973435	-	LOC110375471	uncharacterized LOC110375471	
2	NC_087121.1	3992033	3994408	+	LOC126055971	uncharacterized LOC126055971	
2	NC_087121.1	4002116	4149758	+	LOC110375954	voltage-dependent T-type calcium channel subunit alpha-1G	
2	NC_087121.1	6627440	6632121	+	LOC135117972	uncharacterized LOC135117972	
2	NC_087121.1	6633716	6642064	+	LOC110376138	diacylglycerol kinase epsilon	
2	NC_087121.1	6642622	6651106	-	LOC110376276	mitochondrial dicarboxylate carrier	
2	NC_087121.1	6649684	6651970	+	Elp6	Elongator complex protein 6	
2	NC_087121.1	6653206	6671915	+	LOC110376105	kinesin-like protein KIF19	
2	NC_087121.1	6672231	6688522	-	LOC110376163	pH-sensitive chloride channel 2	
2	NC_087121.1	6688382	6691678	-	LOC110376095	transducin beta-like protein 3	
2	NC_087121.1	6691795	6692946	+	LOC110376340	transmembrane protein 186	
2	NC_087121.1	6693044	6696010	+	LOC135117988	uncharacterized LOC135117988	
2	NC_087121.1	6696024	6697336	-	LOC135117984	putative nuclease HARBI1	
2	NC_087121.1	6697621	6701597	-	LOC110376193	dual specificity mitogen-activated protein kinase kinase 4	
2	NC_087121.1	6702136	6704686	+	LOC110376267	DNA-directed RNA polymerase I subunit RPA43	
2	NC_087121.1	6706824	6708327	+	LOC110376302	small ribosomal subunit protein uS7m	
2	NC_087121.1	6715967	6741543	+	LOC110375993	phospholipid-transporting ATPase ABCA3	
2	NC_087121.1	6741170	6764709	-	LOC110376003	phospholipid-transporting ATPase ABCA1	
2	NC_087121.1	6766869	6776563	+	LOC110375647	ubiquitin carboxyl-terminal hydrolase 31	
2	NC_087121.1	6778146	6783101	-	LOC110377503	uncharacterized LOC110377503	
2	NC_087121.1	6783647	6819922	+	LOC110375548	protein yellow	
2	NC_087121.1	6823287	6841149	+	LOC110375518	protein yellow	
2	NC_087121.1	6841269	6843221	-	LOC110375358	ATP synthase subunit O, mitochondrial	
2	NC_087121.1	6843847	6849475	-	LOC110375580	UPF0764 protein C16orf89 homolog	
2	NC_087121.1	6850173	6858040	+	LOC110377298	protein yellow	
2	NC_087121.1	6858580	6861839	+	LOC110377309	protein yellow	
2	NC_087121.1	6862124	6865299	+	LOC110375349	protein yellow	
2	NC_087121.1	6865424	6873166	-	LOC110375339	uncharacterized LOC110375339	

2	NC_087121.1	6873554	6906013	+	LOC110376662	uncharacterized LOC110376662	
2	NC_087121.1	6894050	6895465	-	LOC135118013	uncharacterized LOC135118013	
2	NC_087121.1	6906619	6908906	+	LOC110377132	L-xylulose reductase	
2	NC_087121.1	6909347	6910817	+	LOC110377141	D-erythrulose reductase	
2	NC_087121.1	6910821	6919174	-	LOC110377122	casein kinase I	
2	NC_087121.1	6920527	6936164	-	LOC110376783	class A basic helix-loop-helix protein 15	rs2P6931787 (GWAS)
2	NC_087121.1	6939491	6950267	+	LOC135118004	uncharacterized LOC135118004	
2	NC_087121.1	6950268	6952564	-	LOC110377015	uncharacterized LOC110377015	
2	NC_087121.1	6953213	6955408	+	LOC110375774	uncharacterized LOC110375774	
2	NC_087121.1	6955182	6957571	-	LOC110375765	uncharacterized protein	
2	NC_087121.1	6982067	6996873	+	LOC110377192	uncharacterized LOC110377192	
2	NC_087121.1	7031730	7033701	+	LOC110375944	ribosomal L1 domain-containing protein 1	
2	NC_087121.1	7033612	7074133	-	Nbs	nbs	
2	NC_087121.1	7036413	7048435	+	Rempa	reduced mechanoreceptor potential A	
2	NC_087121.1	7044142	7050355	-	LOC110377230	uncharacterized LOC110377230	
2	NC_087121.1	7046617	7050185	-	LOC126056560	uncharacterized LOC126056560	
2	NC_087121.1	7050727	7055693	+	LOC110375709	translation factor GUF1 homolog, mitochondrial	
2	NC_087121.1	7055914	7060358	-	LOC110375720	WD repeat domain phosphoinositide-interacting protein 3	
2	NC_087121.1	7061101	7061853	-	LOC110376651	ragulator complex protein LAMTOR2 homolog	
2	NC_087121.1	7063283	7065678	-	LOC110376535	protein SDA1 homolog	
2	NC_087121.1	7066134	7069778	+	Kdelr	KDEL receptor	
2	NC_087121.1	7070997	7073468	-	LOC110377236	DNA polymerase subunit gamma-2, mitochondrial	
2	NC_087121.1	7079923	7086476	+	LOC110377421	aldo-keto reductase family 1 member B1	
2	NC_087121.1	7087937	7092530	+	LOC135118725	uncharacterized LOC135118725	
2	NC_087121.1	7096825	7098942	+	LOC110383041	putative nuclease HARBI1	
2	NC_087121.1	7098877	7100974	-	LOC110383055	uncharacterized LOC110383055	
2	NC_087121.1	7106324	7112865	+	LOC135118029	aldo-keto reductase family 1 member B1-like	
2	NC_087121.1	7114449	7161492	+	LOC135118726	uncharacterized LOC135118726	
2	NC_087121.1	7182091	7189907	+	LOC135118033	uncharacterized LOC135118033	
2	NC_087121.1	7190014	7207134	-	LOC110377596	chaoptin	
2	NC_087121.1	13805097	13805564	-	LOC126055221	uncharacterized LOC126055221	
2	NC_087121.1	13848977	13850417	+	LOC126055232	uncharacterized LOC126055232	
2	NC_087121.1	13851523	13852393	-	LOC135118818	uncharacterized LOC135118818	
2	NC_087121.1	13871344	13913818	+	LOC135116632	lethal(2) giant larvae protein homolog 1-like	
2	NC_087121.1	13916795	13922310	+	LOC135118821	oocyte zinc finger protein XlCOF6.1-like	
2	NC_087121.1	13923168	13930539	-	LOC135118822	coiled-coil domain-containing protein 40-like	
2	NC_087121.1	13930526	13935849	+	LOC135118766	intraflagellar transport protein 56-like	
2	NC_087121.1	13938182	13940505	-	LOC135118884	cuticle protein 21-like	
2	NC_087121.1	13943292	13946723	-	LOC110379225	sodium/potassium-transporting ATPase subunit alpha	
2	NC_087121.1	13949681	13964220	-	LOC135118493	WD repeat-containing protein 81-like	
2	NC_087121.1	13964580	13966789	-	LOC135118496	uridine 5'-monophosphate synthase-like	
2	NC_087121.1	13974042	13996862	+	LOC135118767	zinc finger protein 436-like	
2	NC_087121.1	13998256	14004246	-	LOC110379415	zinc finger protein 689	
2	NC_087121.1	14009513	14020390	-	LOC110378434	zinc finger protein with KRAB and SCAN domains 7	
2	NC_087121.1	14061235	14062661	-	LOC135118505	uncharacterized LOC135118505	
2	NC_087121.1	14062827	14065091	+	LOC110378449	actin-5C	
2	NC_087121.1	14066584	14095088	-	LOC110377798	klaroid protein	
2	NC_087121.1	14095427	14116964	+	LOC135118502	Fanconi anemia group I protein homolog	rs2P14104337 (Upper boundary of the QTL)
2	NC_087121.1	14130057	14130874	+	LOC135118389	uncharacterized LOC135118389	

2	NC_087121.1	14148376	14219924	-	LOC135118768	uncharacterized LOC135118768	
2	NC_087121.1	14180157	14180786	-	LOC135118507	uncharacterized LOC135118507	
2	NC_087121.1	14224577	14226272	+	LOC135118508	NADH dehydrogenase [ubiquinone] 1 beta subcomplex subunit 10-like	
2	NC_087121.1	14228103	14245459	-	LOC135118510	meiosis regulator and mRNA stability factor 1-like	
2	NC_087121.1	14246107	14256116	+	LOC135118511	26S proteasome non-ATPase regulatory subunit 12-like	
2	NC_087121.1	14256507	14260129	+	LOC110375695	uncharacterized LOC110375695	
2	NC_087121.1	14260223	14270956	-	LOC135118513	inositol polyphosphate 5-phosphatase E-like	
2	NC_087121.1	14273372	14280241	-	LOC135118514	uncharacterized LOC135118514	
2	NC_087121.1	14283177	14316851	+	LOC135116537	putative inorganic phosphate cotransporter	
2	NC_087121.1	14316842	14327874	-	LOC110377209	RAD50-interacting protein 1	
2	NC_087121.1	14328104	14332895	+	LOC135118524	lysophospholipid acyltransferase 7-like	
2	NC_087121.1	14332687	14348594	-	LOC110377700	keratin%2C type I cytoskeletal 9	
13	NC_087132.1	3867903	3872274	-	LOC110384348	tyrosine-protein kinase transmembrane receptor Ror	
13	NC_087132.1	3873100	3874636	-	LOC110384349	thyrostimulin beta-5 subunit	
13	NC_087132.1	3878673	3880298	+	LOC110384456	uncharacterized LOC110384456	
13	NC_087132.1	3880709	3892911	+	LOC110384457	leucine-rich repeat-containing protein 40	
13	NC_087132.1	3902663	3920466	+	LOC110384403	uncharacterized LOC110384403	
13	NC_087132.1	3921226	3923037	-	LOC110384417	pro-resilin	
13	NC_087132.1	3923709	3925558	+	LOC110384401	protein SCO1 homolog, mitochondrial	
13	NC_087132.1	3925686	3930172	-	LOC135117704	zinc finger protein 660-like	
13	NC_087132.1	3930215	3935858	-	LOC110384400	zinc finger protein 112	
13	NC_087132.1	3939433	3940993	+	LOC110384384	mitochondrial basic amino acids transporter	
13	NC_087132.1	3941409	3951540	-	LOC110384382	kelch-like protein 10	
13	NC_087132.1	3951663	3966400	-	Lkrsdh	Lysine ketoglutarate reductase/saccharopine dehydrogenase	
13	NC_087132.1	3956460	3956531	+	Trnae-uuc-5	transfer RNA glutamic acid (anticodon UUC)	
13	NC_087132.1	3968177	3988811	+	LOC110384363	ethanolaminephosphotransferase 1	
13	NC_087132.1	3990213	4122492	-	LOC110384364	RNA polymerase II transcriptional coactivator	
13	NC_087132.1	3991445	3993013	+	LOC110384344	uncharacterized LOC110384344	
13	NC_087132.1	3992985	4003965	-	LOC110384342	formin-2	
13	NC_087132.1	4014340	4038780	+	LOC110384404	uncharacterized LOC110384404	
13	NC_087132.1	4046670	4101214	-	LOC110384339	hemicentin-2	
13	NC_087132.1	4142457	4154672	+	LOC110384365	esterase FE4	
13	NC_087132.1	4156145	4158871	-	LOC110384366	splicing factor U2af 38 kDa subunit	
13	NC_087132.1	4159329	4178569	+	LOC110384360	multidrug resistance-associated protein 1	rs13P4171996 (GWAS)
13	NC_087132.1	4160534	4162466	+	LOC126055707	uncharacterized LOC126055707	
13	NC_087132.1	4180513	4216417	+	LOC110384372	multidrug resistance-associated protein 1	
13	NC_087132.1	4185164	4187608	+	LOC126054123	uncharacterized LOC126054123	
13	NC_087132.1	4187552	4190243	-	LOC126053835	putative nuclease HARBI1	
13	NC_087132.1	4217425	4219118	+	LOC135117737	uncharacterized LOC135117737	
13	NC_087132.1	4222317	4241801	-	LOC110384329	A-kinase anchor protein 200	
13	NC_087132.1	4242968	4249693	+	LOC110384358	transcription factor AP-4	
13	NC_087132.1	4253163	4259663	+	LOC110384443	serpin B8	
13	NC_087132.1	4259539	4260805	-	LOC110384445	male-enhanced antigen 1	
13	NC_087132.1	4262496	4265223	+	Pcmt	Protein-L-isoaspartate (D-aspartate) O-methyltransferase	
13	NC_087132.1	4265133	4269762	-	LOC110384442	DNA repair endonuclease XPF	
13	NC_087132.1	4271626	4289596	+	LOC110384357	hrp65 protein	
13	NC_087132.1	4290454	4294932	-	LOC110371640	lipase member H	
13	NC_087132.1	4296360	4352480	-	LOC110371612	protein turtle	
13	NC_087132.1	4355561	4363349	+	LOC110371642	polyphosphoinositide phosphatase	

13	NC_087132.1	4365817	4372347	+	LOC110371599	synaptojanin-1	
13	NC_087132.1	4373529	4377186	-	LOC110371665	cytochrome c oxidase subunit 7A-related protein, mitochondrial	
13	NC_087132.1	4387966	4395393	-	LOC110371623	polyubiquitin	
13	NC_087132.1	4395852	4396899	+	LOC110371622	ubiquitin-ribosomal protein eL40 fusion protein	
13	NC_087132.1	4397837	4405650	-	LOC110371621	WD repeat-containing and planar cell polarity effector protein fritz homolog	
13	NC_087132.1	4424151	4512064	+	LOC110371637	pyrokinin-1 receptor	
13	NC_087132.1	6386520	6406627	-	LOC110376158	uncharacterized LOC110376158	
13	NC_087132.1	6415823	6421755	-	LOC110376146	lipase member H	
13	NC_087132.1	6426243	6494802	-	LOC110376155	uncharacterized LOC110376155	
13	NC_087132.1	6495673	6498324	+	LOC110370743	palmitoyltransferase ZDHHC23-A	
13	NC_087132.1	6498206	6504116	-	LOC110370718	girdin	
13	NC_087132.1	6504312	6505855	+	LOC110370687	post-GPI attachment to proteins factor 2	
13	NC_087132.1	6505326	6509046	-	LOC110370685	L-2-hydroxyglutarate dehydrogenase, mitochondrial	
13	NC_087132.1	6509508	6511844	+	LOC110370686	modifier of mdg4	
13	NC_087132.1	6512563	6516472	+	LOC135117716	transcription activator GAGA-like	
13	NC_087132.1	6519760	6527828	+	LOC110370673	aminoacylase-1	
13	NC_087132.1	6529966	6542161	+	LOC110370749	aminoacylase-1	
13	NC_087132.1	6547003	6569086	+	LOC110370671	protein dissatisfaction	
13	NC_087132.1	6569697	6571947	-	LOC110370737	cysteine and histidine-rich domain-containing protein morgana	
13	NC_087132.1	6572481	6601142	+	LOC110370670	WD repeat and FYVE domain-containing protein 3	
13	NC_087132.1	6601312	6625598	-	LOC110370669	protein numb	
13	NC_087132.1	6625622	6635698	+	LOC110370752	xanthine dehydrogenase	
13	NC_087132.1	6626514	6628305	+	LOC110370753	cytochrome c oxidase assembly protein COX20, mitochondrial	
13	NC_087132.1	6630578	6630650	+	Trnak-cuu-7	transfer RNA lysine (anticodon CUU)	
13	NC_087132.1	6637884	6645891	+	LOC110370678	xanthine dehydrogenase	
13	NC_087132.1	6645966	6655706	-	Tango1	Transport and Golgi organization 1	
13	NC_087132.1	6656475	6661979	+	LOC110370751	uncharacterized LOC110370751	
13	NC_087132.1	6700310	6784224	+	LOC110370738	protein O-mannosyl-transferase TMTC2	rs13P6678940 (GWAS)
